# Unveiling the Metabolic Effects of Glycomacropeptide

**DOI:** 10.3390/ijms22189731

**Published:** 2021-09-08

**Authors:** Maria João Pena, Raquel Costa, Ilda Rodrigues, Sandra Martins, João Tiago Guimarães, Ana Faria, Conceição Calhau, Júlio César Rocha, Nuno Borges

**Affiliations:** 1 Departamento de Biomedicina, Unidade de Bioquímica, Faculdade de Medicina, Universidade do Porto, 4200-319 Porto, Portugal; up200702810@edu.med.up.pt (M.J.P.); mrcosta@med.up.pt (R.C.); irodrigues@med.up.pt (I.R.); jtguimar@med.up.pt (J.T.G.); 2 i3S—Instituto de Investigação e Inovação em Saúde, Universidade do Porto, 4200-135 Porto, Portugal; 3 Department of Clinical Pathology, São João Hospital Centre, 4200-319 Porto, Portugal; smvrmartins@gmail.com; 4 Instituto de Saúde Pública, Universidade do Porto, 4050-091 Porto, Portugal; 5 Nutrition & Metabolism, NOVA Medical School, Faculdade de Ciências Médicas, Universidade NOVA de Lisboa, 1169-056 Lisboa, Portugal; ana.faria@nms.unl.pt (A.F.); ccalhau@nms.unl.pt (C.C.); rochajc@nms.unl.pt (J.C.R.); 6 CINTESIS—Centre for Health Technology and Services Research, 4200-450 Porto, Portugal; 7 CHRC—Comprehensive Health Research Centre, Universidade NOVA de Lisboa, 1169-056 Lisboa, Portugal; 8 Unidade Universitária Lifestyle Medicine da José de Mello Saúde by NOVA Medical School, 1169-056 Lisboa, Portugal; 9 Reference Centre of Inherited Metabolic Diseases, Centro Hospitalar Universitário de Lisboa Central, 1169-045 Lisboa, Portugal; 10 Faculdade de Ciências da Nutrição e Alimentação, Universidade do Porto, 4150-180 Porto, Portugal

**Keywords:** glycomacropeptide, amino acids, intact protein, metabolism, GLP-1, phenylketonuria

## Abstract

For many years, the main nitrogen source for patients with phenylketonuria (PKU) was phenylalanine-free amino acid supplements. Recently, casein glycomacropeptide (GMP) supplements have been prescribed due to its functional and sensorial properties. Nevertheless, many doubts still persist about the metabolic effects of GMP compared to free amino acids (fAA) and intact proteins such as casein (CAS). We endeavour to compare, in rats, the metabolic effects of different nitrogen sources. Twenty-four male Wistar rats were fed equal energy density diets plus CAS (control, *n* = 8), fAA (*n* = 8) or GMP (*n* = 8) for 8 weeks. Food, liquid intake and body weight were measured weekly. Blood biochemical parameters and markers of glycidic metabolism were assessed. Glucagon-like peptide-1 (GLP-1) was analysed by ELISA and immunohistochemistry. Food intake was higher in rats fed CAS compared to fAA or GMP throughout the treatment period. Fluid intake was similar between rats fed fAA and GMP. Body weight was systematically lower in rats fed fAA and GMP compared to those fed CAS, and still, from week 4 onwards, there were differences between fAA and GMP. None of the treatments appeared to induce consistent changes in glycaemia, while insulin levels were significantly higher in GMP. Likewise, the production of GLP-1 was higher in rats fed GMP when compared to fAA. Decreased urea, total protein and triglycerides were seen both in fAA and GMP related to CAS. GMP also reduced albumin and triglycerides in comparison to CAS and fAA, respectively. The chronic consumption of the diets triggers different metabolic responses which may provide clues to further study potential underlying mechanisms.

## 1. Introduction

Phenylketonuria (PKU, OMIM # 261600) stems from the body’s inability to metabolize the essential amino acid phenylalanine (Phe) to tyrosine (Tyr) due to deficient activity of phenylalanine hydroxylase (PAH) with resultant cognitive impairment [1]. This can be avoided with a Phe-restricted diet in combination with protein substitutes and special low protein foods [2]. However, the taste of Phe-free amino acid supplements hampers adherence [3,4,5]. In recent years, new modalities of treatment have arisen, namely glycomacropeptide (GMP) and its commercial formulations, casein glycomacropeptide supplements [6].

GMP is a whey-based bioactive peptide derived from the hydrolysis of milk κ-casein that presents beneficial properties on human health but whose formulations are not completely devoid of Phe [7]. According to the available data from clinical studies, GMP presents a better taste and improves satiety and nitrogen retention. In preclinical studies with PKU mice, there is evidence that GMP reduces Phe in the brain, improves bone health and acts as prebiotic [8].

Free AA (fAA), typically present in Phe-free amino acid supplements. presents faster absorption kinetics that distinguishes them from intact proteins, which may affect the efficiency of nitrogen utilization because amino acids are released at different rates in the blood stream. This physiology of absorption may disrupt whole-body metabolism [9]. Studies using Phe-free amino acid supplements applying a prolonged-release technology showed that these formulations present better organoleptic characteristics and can support a more physiological amino acid absorption, therefore improving metabolism [4,5]. Regarding GMP, a very recent study from Daly et al. showed that casein glycomacropeptide supplements seem to have a similar kinetics absorption as Phe-free amino acid supplements [10]. However, these data are still poorly understood.

Among the metabolic effects are the impact on glycidic metabolism markers, particularly on blood glucose, insulin, and glucagon-like peptide-1 (GLP-1) secretion. Amino acids act as important triggers of insulin secretion, mainly branched-chain amino acids, of which leucine (Leu) has a more pronounced effect [11]. In addition, we know that nutrient intake is a major stimulus for GLP-1 release from the L-cells which are expressed in the gastrointestinal tract [12]. The anatomical region with the highest amount of GLP-1 is the distal ileum, both in humans and rats [13,14]. Beyond its role as an incretin, GLP-1 is a pleiotropic hormone with a multitude of metabolic functions [13].

Mönch et al. reported that the bolus administration of Phe-free amino acid supplements increases the urinary excretion of nitrogen when the fast increase in blood amino acids exceeds the capacity of anabolism to incorporate them into nascent proteins [15]. Still, Weigel et al. observed a significantly higher insulin peak at 30 min after the intake of Phe-free amino acid supplements [16]. In accordance, a study from our group showed that the intake of one Phe-free amino acid supplement by wild-type rats led to lower glucose levels when compared to intact protein. A putative explanation was the insulinogenic properties of some amino acids, especially Leu, that is available in large amounts in Phe-free amino acid supplements, as well as the incretin axis that might have contributed to this finding [17].

Taking all of this into account, it is likely that these nitrogen sources could yield different metabolic responses. Therefore, our aim was to evaluate the chronic effect of CAS, fAA and GMP on metabolic parameters, especially on glycidic metabolism biomarkers by feeding young and healthy Wistar rats with those diets for 8 weeks.

## 2. Results

### 2.1. Food Intake, Fluid Intake and Body Weight

**Figure 1:**
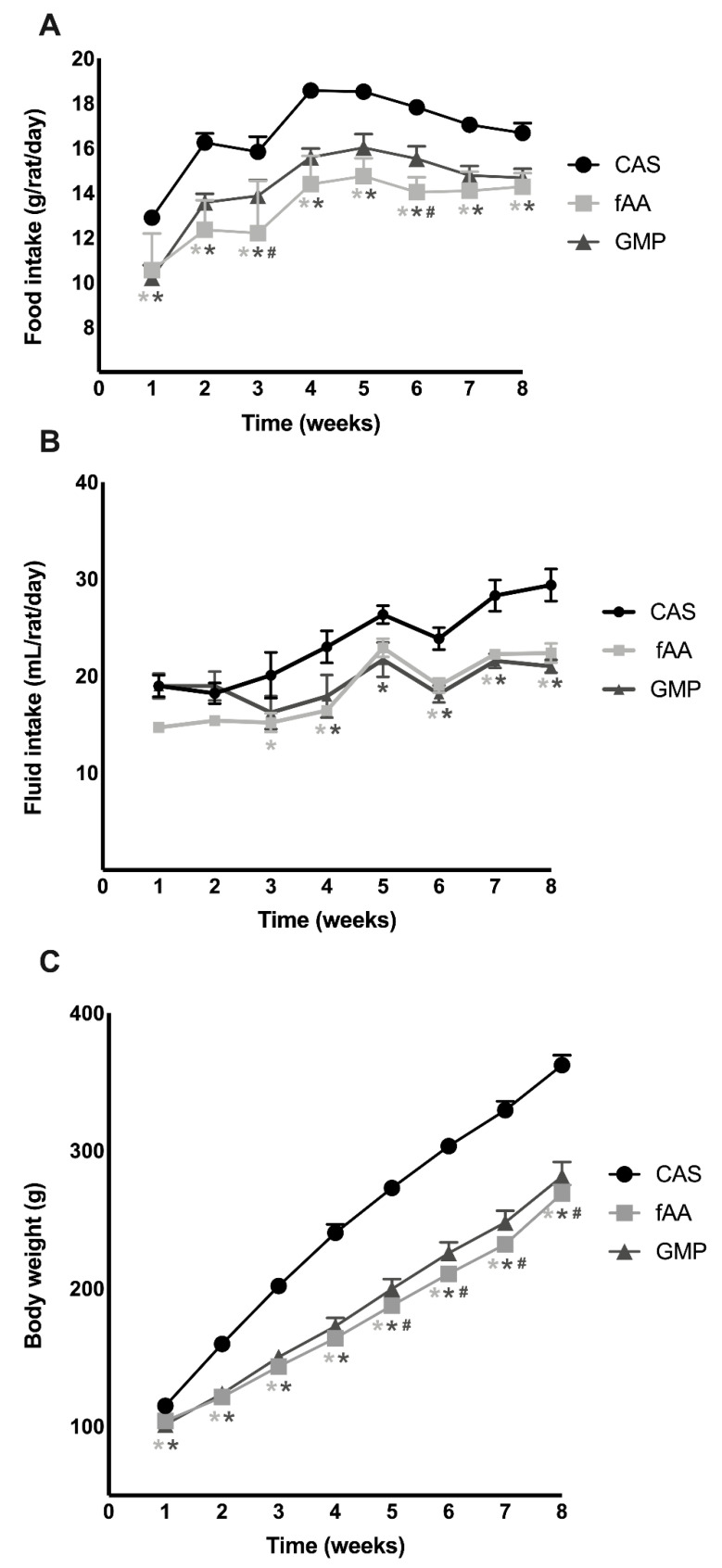
Monitoring of food intake (**A**), fluid intake (**B**) and body weight (**C**) of Wistar rats over the 8-week feeding period. Data are presented as mean ± SEM (*n* = 8/group). Two-way ANOVA with repeated measures followed by Bonferroni’s post-hoc test was performed. * *p* < 0.05 vs. CAS; # *p* < 0.05 vs. fAA. CAS: casein; fAA: free amino acids; GMP: glycomacropeptide.

Apart from weeks 3 (*p* = 0.0092) and 6 (*p* = 0.0218), no differences were observed in food intake between fAA and GMP. On the other hand, food intake was higher in rats fed the CAS diet when compared to fAA or GMP throughout the weeks of treatment (*p* < 0.05) (Figure 1A).

Fluid intake was also similar between animals consuming fAA and those with GMP (Figure 1B). From week 3 onwards, rats fed CAS had higher fluid ingestion compared to the other experimental groups (*p* < 0.05).

In agreement with the reduced food intake measured throughout an 8-week period, rats fed fAA and GMP showed a lower body weight compared to those rats fed CAS (*p* < 0.05). Additionally, from week 4 onwards, rats fed fAA presented lower body weight compared to rats fed GMP (*p* < 0.0.5) (Figure 1C).

### 2.2. Markers of Glucose Metabolism

**Figure 2:**
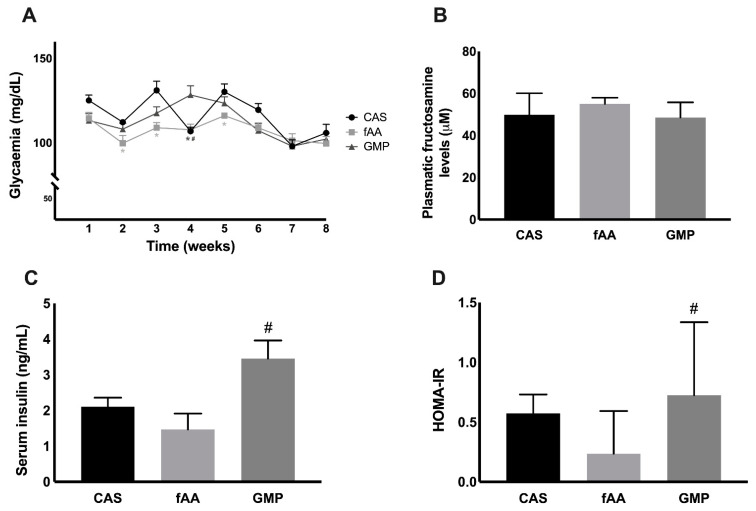
Glycaemia (**A**) of Wistar rats over the 8-week feeding period. Data are presented as mean ± SEM (*n* = 8/group). Two-way ANOVA with repeated measures followed by Bonferroni’s post-hoc test was performed. * *p* < 0.05 vs. CAS; # *p* < 0.05 vs. fAA. Plasmatic fructosamine (**B**) in the 3 study groups. Data are presented as mean ± SEM (*n* = 8/group). One-way ANOVA with Bonferroni’s post-hoc test was performed. Serum insulin (**C**) in the 3 study groups. Data are presented as mean ± SEM (*n* = 6/group). One-way ANOVA with Bonferroni’s post-hoc test was performed. # *p* < 0.05 vs. fAA. HOMA-IR (**D**) in the 3 study groups. Data are presented as median (P25-P75) (*n* = 6/group). Kruskal–Wallis with Dunn’s post-hoc test was performed. # *p* < 0.05 vs. fAA. CAS: casein; fAA: free amino acids; GMP: glycomacropeptide; HOMA-IR: homeostasis model assessment for insulin resistance.

To further understand the effect of the different diets on markers of glucose metabolism, glycaemia, fructosamine, insulin levels and homeostasis model assessment for insulin resistance (HOMA-IR) were determined. With regard to glycaemia, at weeks 2 (*p* = 0.0446), 3 (*p* < 0.0001) and 5 (*p* = 0.0184), significant changes were found between fAA and CAS. Still, at week 4, glycaemia was significantly different between GMP and CAS (*p* = 0.0001) and GMP and fAA (*p* = 0.0003) (Figure 2A). When it comes to fructosamine, no differences were seen between the groups (Figure 2B) at the end of the treatment. On the other hand, after 8 weeks, animals treated with GMP presented higher insulin levels in comparison with those treated with fAA (*p* = 0.0127) (Figure 2C). The HOMA-IR was calculated, and a significant difference was observed between GMP and fAA (*p* = 0.0305) (Figure 2D).

### 2.3. Local and Systemic GLP-1

**Figure 3:**
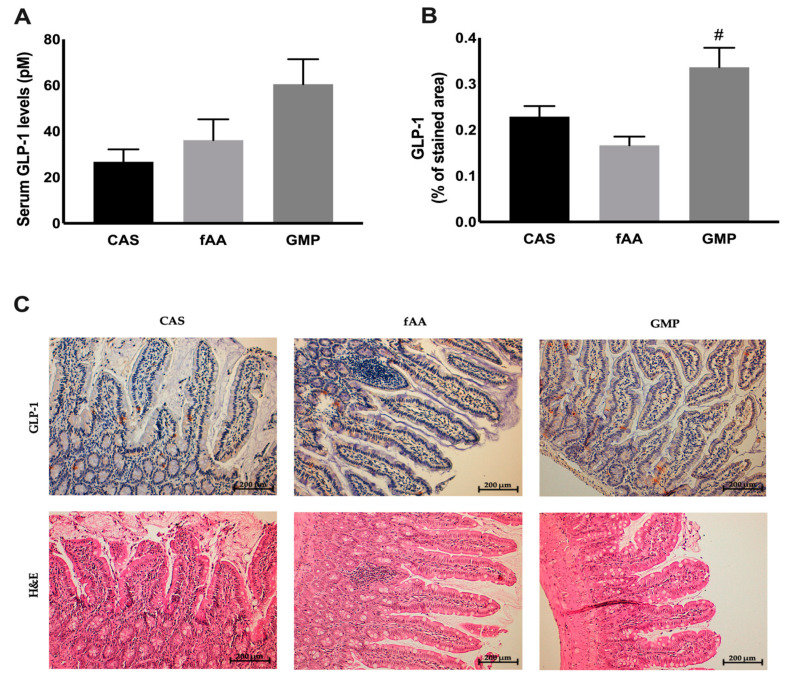
Serum GLP-1 levels (**A**) in the 3 study groups. Data are presented as mean ± SEM (*n* = 4–7/group). One-way ANOVA with Bonferroni’s post-hoc test was performed. GLP-1 (% of stained area) (**B**) in the 3 study groups. Data are presented as mean ± SEM (*n* = 34–39 images/group). One-way ANOVA with Bonferroni’s post-hoc test was performed. # *p* < 0.05 vs. fAA. Ileum GLP-1 IHC and H&E images (**C**). CAS: casein; fAA: free amino acids; GLP-1: glucagon-like peptide-1; GMP: glycomacropeptide; H&E: hematoxylin and eosin; IHC: immunohistochemistry.

At the endpoint of the study, GLP-1 was explored. Circulating GLP-1 was not different among groups, although a tendency for greater levels in the GMP group was observed (Figure 3A). Regarding the production of GLP-1 by intestinal enteroendocrine L-cells, a statistically significant difference was found between GMP and fAA, with a higher production in animals fed GMP (*p* < 0.0001) (Figure 3B). Figure 3C shows representative images of Immunohistochemistry (IHC) and Hematoxylin and Eosin (H&E).

### 2.4. Biochemical Markers

Table 1 depicts the serum biochemical markers measured at the end of the study. Considerable differences were found in alkaline phosphatase (ALP) in the GMP group towards fAA (*p* = 0.0156). When it comes to nutritional markers, it is worthwhile to mention the lower levels of urea in the rats fed fAA (*p* = 0.0143) and GMP (*p* = 0.0161) when compared to CAS. The same was observed in total protein for fAA vs. CAS (*p* < 0.0001) and for GMP vs. CAS (*p* = 0.0001). In addition, GMP reduced albumin concentration when compared to CAS (*p* = 0.0247). The levels of triglycerides were decreased in animals either fed fAA vs. CAS (*p* = 0.0051) or GMP vs. CAS (*p* = 0.0150). Total cholesterol was significantly lower in rats fed GMP relative to fAA (*p* = 0.0348). Still, iron was significantly reduced in fAA against CAS (*p* = 0.0459). There was a tendency to decreased pro-inflammatory cytokines in rats fed GMP, but this did not reach statistical significance.

## 3. Discussion

In this study, we sought to understand the metabolic impact of different forms of nitrogen, CAS, a standard of an intact protein, fAA that are present in Phe-free amino acid supplements, and GMP, a whey-based protein, in post-weaning Wistar rats, for 8 weeks. We used isoenergetic standard rodent diets in which intact protein was replaced by fAA and GMP, and whose amino acid profile of fAA and GMP was similar. All diets contained 20% of protein expressed in distinctive nitrogen sources.

Our results revealed that, except for weeks 3 and 6, food intake was significantly lower in rats fed experimental diets when compared to the CAS diet, and the organoleptic features (odour and taste) of those diets mainly consisting of fAA and GMP plus amino acids might have been responsible for the differences found. Another important point is that dairy whey protein is thought to be more satiating than other protein sources [18]. Therefore, as a whey-based protein, the effect of GMP on satiety has been subjected to study, but conflicting results exist. Some studies performed in PKU patients showed that GMP appeared to have a satiating effect [8], whereas other studies in this population did not follow suit [19]. Furthermore, studies carried out in Wistar rats [20] and healthy subjects [18,21] failed to find an effect of GMP on satiety. With respect to fluid intake, rats fed experimental diets had significantly lower consumption during the experimental period. Consequently, variations in body weight were seen because of different food and fluid intakes.

Regarding glycaemia, significant but not consistent differences were observed between some of the groups. Therefore, overall, none of the treatments appeared to induce a systematic change in glycaemia. Fructosamine is a glycated protein that reflects short- term (2–3 weeks) changes in glucose control when compared to glycated haemoglobin (HbA1c) because albumin has a shorter half-life of approximately 20 days [22]. No differences were found in fructosamine between the groups. On the other hand, the insulin levels were higher in rats fed GMP in comparison with fAA, in contrast to what might be expected, that was, insulin levels higher in rats fed fAA [23]. It is possible that the post-absorptive levels of amino acids were lower. Theoretically, Leu and the mammalian target of rapamycin (mTOR) pathway could affect insulin secretion and glucose homeostasis by exerting influence in pancreatic islet β-cells and tissues such as liver and muscles [24]. The GMP diet contained similar amounts of Leu to those from fAA, with the exception that around half of the amount came from intact protein and the other half from free amino acids. Furthermore, the other branched-chain amino acids (isoleucine and valine) are also insulinogenic [11], and the amounts only came from original GMP without additional amino acids. Insulin levels were only measured at the end and not during the experiment which could provide further information as to the efficacy of GMP to stimulate insulin release. When it comes to HOMA-IR, a surrogate measure of insulin sensitivity, a significant difference was observed between GMP and fAA, indicating that animals fed fAA were 3-fold less insulin sensitive than those rats fed GMP. At the intestinal level, the GMP diet induced a significant production of GLP-1 by L-cells when compared to fAA, and despite the lack of significance, a tendency for systemic higher levels were also observed. A short-term study addressing the impact of consuming different drink mixtures containing either GMP or fAA showed that neither of them elicit a significant effect on GLP-1 [25].

Changes in nutritional status markers and ALP were observed in rats treated with fAA and GMP diets. The lower levels of these parameters could reflect the reduced food intake. Additionally, since animals were treated from weaning, these findings could be explained by adaptative responses to diets with distinct nitrogen sources. The reduced levels of triglycerides and total cholesterol in rats fed GMP may be due its hypolipidemic activity [26]. The liver has a significant role in inflammation processes and this dynamic is influenced by inflammatory mediators and plasma proteins [27]. Serum pro-inflammatory cytokines (IL-1β, IL-6 and TNF-α) were determined. Despite the absence of significant differences among groups, a tendency to a lower inflammatory tone was shown in rats fed GMP. Studies in the PKU mouse model showed that GMP reduced inflammatory markers in comparison with fAA and CAS [28,29]. A study aiming at evaluating the effect of intake of different nitrogen sources, CAS or fAA, by adult mice at steady state and during colitis development, reported that mice fed fAA had increased levels of inflammatory mediators in the small and large intestines [30]. In addition, each of these cytokines offers a “half angel—half devil” facet and none can be simply categorized either “pro” or “anti” [31] since several parameters can interfere with their action.

The absorption kinetics between proteins, peptides, and amino acids is a regulating factor for postprandial metabolism both in human and animal models [32], being faster when single amino acids are ingested compared with an intact protein [33]. In the context of PKU patients, it is important to bear in mind that L-AA are prescribed in high doses, as well as the industry tailor-makes their amino acid profile to meet the patients’ daily requirements. Herein, GMP is comprised of primarily an intact protein supplemented with amino acids. The bioavailability of GMP and its potential to influence glycaemic control and related metabolism is still poorly understood. A study from Daly et al. showed that casein glycomacropeptide supplements seems to have a similar kinetics absorption of Phe-free amino acid supplements [10]. In this context, additional studies are required to gain a more integrated insight into the absorption kinetics of GMP that might aid understanding its metabolic impact.

This study has some limitations. We used an animal model without disease instead of a mouse model of PKU. To avoid stress in the rats, we did not opt into utilizing oral gavage and thus it was difficult to control the amount of food each rat consumed.

Future intervention clinical studies will in fact be necessary to refine the data obtained in this study. We are interested in going further and understanding how different nitrogen sources might shape the gut microbiota, firstly in rats and afterwards in PKU patients. Data from clinical studies show that the type of protein substitute affects Tyr degradation differently, with Phe-free amino acid supplements providing less bioavailable Tyr due in part to a higher degree of Tyr degradation by the resident gut bacteria and also due to less frequent intake throughout the day when compared to casein glycomacropeptide supplements [34]. Additionally, a reduction was observed in plasma deoxycarnitine in PKU patients that could be related to their documented reduced carnitine biosynthesis [35]. We know from the literature that plasma levels of trimethylamine N-oxide (TMAO), an active molecule generated by the gut microbiota, have been correlated with the risk of cardiovascular disease [36]. In PKU patients, the dysbiosis can be related to the underlying disease or the diet itself [37], but so far, the evidence is insufficient. These are important angles for further studies.

## 4. Materials and Methods

### 4.1. Animals and Housing

Twenty-four male Wistar rats from weaning (62 g) were obtained from Charles River Laboratories (Barcelona, Spain). Upon arrival, rats were housed 2 per cage and kept in animal facilities under controlled conditions of light (12 h light–dark cycle), temperature (22–24 °C), humidity (55 ± 10%) and with 15 air changes/h. The animals were given free access to food and water ad libitum for 1 week to acclimatise to the new environment. All animal experiments were in accordance with the European Union guidelines (Directive 2010/63/EU) and the Portuguese Decree-Law (113/2013) and approved by the Institutional Animal Care and Use Committee of the Faculty of Medicine of the University of Porto (ORBEA_86_2019/1604) in 16 April 2019, and the national authority, Direção Geral de Alimentação e Veterinária (0421/000/000/2021) in 1 February 2021.

### 4.2. Diets

Experimental diets were designed in collaboration with Research Diets, Inc. (New Brunswick, NJ, USA) and prepared using the open standard diet (OSD) with modifications in the nitrogen source (Table 2). Experimental diets containing free amino acids (fAA diet) and glycomacropeptide plus additional amino acids (GMP diet), and control diet comprising whole casein (CAS diet) were isocaloric and similar with regard to all other nutrients. The amino acid profile of the fAA diet matches that of the GMP diet (Table 3). The BiPRO^®^ GMP 9000 from the GMP diet was purchased from Agropur (Appleton, WI, USA) and incorporated into pellets.

### 4.3. Experimental Design

Following the acclimatization period, the 24 rats were randomly divided into 3 groups of 8 animals each: GMP diet, AA diet and CAS diet, the former diet used as control. Animals were maintained on the diets from weaning (21 days) to young adulthood (12 weeks). Body weight was measured once a week using a scale and the remaining food and water in the hopper were reweighed at the same time to map food and fluid intake over time.

At the end of the 8 weeks, animals were deeply anaesthetised with isoflurane 5% (Baxter, IL, USA).

**Figure 4:**
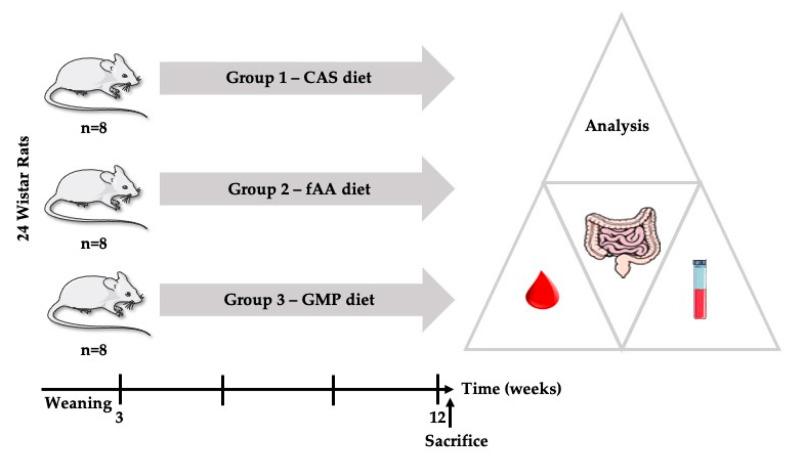
Study design. CAS: casein; fAA: free amino acids; GMP: glycomacropeptide.

The study design is illustrated in Figure 4.

### 4.4. Collection of Blood and Tissues

Blood was drawn from the left ventricle into tubes with or without heparin to obtain plasma and serum, respectively. Aliquots were frozen at −80 °C until further analysis. Then, rats were transcardially perfused with ice-cold isotonic sodium chloride solution. After perfusion, distal ileum was immediately removed and fixed in 10% neutral-buffered formalin.

### 4.5. Glucose, Insulin and Total GLP-1

Glycaemia was measured once a week from the lateral tail vein of the rats with the aid of FreeStyle Precision Neo test strips and device (Abbott Diabetes Care Inc., Maidenhead, UK).

After euthanizing, serum insulin levels were measured using a Rat/Mouse Insulin ELISA kit (EZRMI-13K; Merck Life Science, Darmstadt, Germany) and total GLP-1 was determined using GLP-1 (Total) ELISA kit (Merck Life Science, Darmstadt, Germany), according to the manufacturer’s instructions. HOMA was used as a surrogate measure of insulin resistance using the formula: fasting glucose (mg/dL) × fasting insulin (ng/mL) / 405 [38].

### 4.6. Biochemical Analyses

Routine serum biochemical markers were assessed at the end of the study and performed at the Department of Clinical Pathology, São João Hospital Center, using the AU5400^®^ automated clinical chemistry analyser (Beckman-Coulter^®^, Carnaxide, Portugal). The measurements comprised hepatic function markers (aspartate aminotransferase (AST), alanine aminotransferase (ALT)), renal function markers (creatinine, urea), metabolic/nutritional markers (uric acid, total cholesterol, high-density lipoprotein cholesterol (HDL), low-density lipoprotein cholesterol (LDL), triglycerides, total protein, albumin, transferrin) and other markers (ALP).

Cytokines and leptin were determined using Milliplex^®^ MAP Rat Adipokine Panel—Metabolism Assay, purchased from Arium, Sistemas de Diagnóstico, Lda.

Plasmatic fructosamine was determined using a Rat Fructosamine ELISA kit (ELISAGenie, UK) according to the manufacturer’s instructions.

### 4.7. Immunochemistry for GLP-1

Segments of distal ileum (1 cm of intestine withdrawn above the junction with the caecum) were harvested from all rats and fixed in 10% neutral-buffered formalin for 24 h, dehydrated and embedded in paraffin. The paraffin-embedded tissue sections were cut (3 μm thickness) and mounted on adhesive microscope slides. The sections were deparaffinized in xylene and rehydrated with decreasing concentrations of ethanol (100%, 95% and 70%) and distilled water. Antigen retrieval was performed in 0.01 M sodium citrate buffer with 0.05% Tween 20 (pH 6.0), using a water bath at 98 °C for 10 min and blocked in 3% H_2_O_2_ in PBS. After that, sections were incubated with a blocking solution (Rodent Block M, RBM961G, Biocare Medical, UK) for 30 minutes and then incubated overnight at 4 °C with anti-GLP-1 primary antibody: mouse monoclonal, ab26278; Abcam, Amsterdam, The Netherlands; 1:1500. Subsequently, sections were incubated with secondary antibody at RT for 30 min: goat anti-mouse, ab97021; Abcam, Amsterdam, The Netherlands; 1:200. Then, 30-minute ABC complex incubation was carried out (Vector A and B, Vectastain, Vector Labs, Peterborough, UK). Finally, the sections were treated with diaminobenzidine tetrahydrochloride (DAB) substrate kit (ab64238, Abcam, Amsterdam, The Netherlands). All sections were stained with H&E.

### 4.8. Computerised Image Analysis

Images were acquired with a Nikon Eclipse 50i light microscope (Nikon Corporation Instruments Company, Tokyo, Japan) and analysed using ImageJ^®^ software (National Institute of Health (NIH), MD) with a colour deconvolution plugin which separates the stained area from the initial image. Morphometric analysis was performed in five representative images (200× magnification) from each tissue section. GLP-1 was expressed as the percentage of stained area.

### 4.9. Statistical Analysis

Statistical analysis was performed using GraphPad Prism version 8.0 (GraphPad software, Inc., La Jolla, CA, USA).

The distribution of the variables was checked for normality using Shapiro–Wilk test. Continuous variables are expressed as mean ± standard error of the mean (SEM) or median (P25-P75) as appropriate. For differences in food intake, fluid intake, body weight and glycaemia between the 3 groups throughout the weeks, two-way ANOVA with repeated measures followed by Bonferroni’s post-hoc test was performed. For differences in the remaining parameters, one-way ANOVA with Bonferroni’s post-hoc test was used for normal distribution and Kruskal–Wallis with Dunn’s post-hoc test analysis for non-normal distribution. Significance was set at the level of *p*-value less than 0.05.

## 5. Conclusions

One important message of this study is the lower food intake of rats fed fAA and GMP, and this finding could be associated with a different palatability which is also an issue in PKU patients. In addition, none of the treatments appeared to induce consistent changes in glycaemia, while insulin levels were significantly higher in GMP. Likewise, the production of GLP-1 was higher in rats fed GMP when compared to fAA. Taken together, our findings demonstrate that the type of nitrogen source ingested induces different metabolic responses which may provide clues to further study potential underlying mechanisms. There may be a need to confirm these results and obtain evidence for revision treatment guidelines, at least in some age groups. Furthermore, future studies should go beyond these data and explore the impact on gut microbiota as well.

## Figures and Tables

**Table 1:** Biochemical parameters of the control diet (CAS diet) and the experimental diets (fAA diet and GMP diet).

Parameter	CAS Diet	fAA Diet	GMP Diet
AST (U/L)	89 ± 11	104 ± 16	68 ± 5
ALT (U/L)	17 (15–24)	21 (17–22)	17 (15–20)
ALP (U/L)	146 ± 12	169 ± 11	119 ± 11 #
Creatinine (mg/dL)	0.16 ± 0.01	0.18 ± 0.01	0.17 ± 0.01
Urea (mg/dL)	32 ± 2	24 ± 2 *	24 ± 1 *
Uric acid (mg/dL)	<1.5	<1.5	<1.5
Total cholesterol (mg/dL)	74 ± 5	77 ± 4	61 ± 3 #
LDL-cholesterol (mg/dL)	22 ± 3	24 ± 2	17 ± 2
HDL-cholesterol (mg/dL)	39 ± 4	42 ±2	36 ± 2
Triglycerides (mg/dL)	167 ± 35	55 ± 7 *	68 ± 14 *
Total protein (g/L)	56.5 ± 0.3	51.9 ± 0.3 *	52.1 ± 0.9 *
Albumin (g/L)	28.9 ± 0.5	26.8 ± 0.5	26.1 ± 0.9 *
Iron (µg/dL)	187 (176–209)	162 (151–174) *	177 (161–189)
Transferrin (mg/dL)	175 (167–177)	158 (154–174)	170 (166–175)
Transferrin saturation (%)	78 (74–85)	72 (68–77)	73 (65–82)
Leptin (pg/mL)	687 ± 143	692 ± 194	756 ± 136
IL-1β (pg/mL)	12.0 ± 2.4	5.9 ± 1.5	6.9 ± 1.6
IL-6 (pg/mL)	36 (17–47)	71 (35–165)	20 (10–74)
TNF-α (pg/mL)	1.5 (0.9–2.2)	2.2 (0.4–3.5)	1.0 (0.4–1.3)

Data are presented as mean ± SEM or median (P25-P75) as appropriate (*n* = 4–8/group). One-way ANOVA with Bonferroni’s post-hoc test analysis for normal distribution and Kruskal–Wallis with Dunn’s post-hoc test analysis for non-normal distribution. * *p* < 0.05 vs. CAS; **#** *p* < 0.05 vs. fAA. ALP: alkaline phosphatase; ALT: alanine transaminase; AST: aspartate transaminase; CAS: casein; fAA: free amino acids; GMP: glycomacropeptide; HDL: high-density lipoprotein cholesterol; IL-1β: interleukin-1beta; IL-6: interleukin-6; LDL: low-density lipoprotein cholesterol; TNF-α: tumour necrosis factor-alpha.

**Table 2:** Composition of the control diet (CAS diet) and the experimental diets (L-AA diet and GMP diet).

Ingredients (g/100 g)	CAS Diet	fAA Diet	GMP Diet
Casein	20.0	-	-
BiPRO^®^ GMP	-	-	20.3
Additional L-AA	3.5	20.9	3.2
Protein	20.9	20.9	20.9
CHO	61.9	61.9	61.9
Fat	7.0	7.0	7.0
Fiber	10.0	10.0	10.0
Energy (kcal/g)	107.2	104.6	107.3

CAS: casein; CHO: carbohydrate; fAA: free amino acids; GMP: glycomacropeptide.

**Table 3:** Amino acid profile of the control diet (CAS diet) and the experimental diets (fAA diet and GMP diet).

Amino Acid Profile (g/100 g)	CAS Diet	fAA Diet	GMP Diet *
L-cysteine	0.42	0.02	0.02 (0.02 + 0.00)
L-isoleucine	0.75	1.78	2.11 (2.11 + 0.00)
L-leucine	2.09	1.07	0.94 (0.42 + 0.52)
L-lysine	1.30	1.02	1.21 (1.21 + 0.00)
L-methionine	1.18	0.98	1.04 (0.36 + 0.68)
L-phenylalanine	1.81	1.02	1.02 (0.04 + 0.98)
L-threonine	0.71	2.90	3.43 (3.43 + 0.00)
L-tryptophan	0.39	0.20	0.20 (0.02 + 0.18)
L-valine	0.92	1.57	1.86 (1.86 + 0.00)
L-histidine	0.71	0.28	0.28 (0.02 + 0.26)
L-alanine	0.50	1.04	1.23 (1.23 + 0.00)
L-arginine	0.98	0.43	0.43 (0.04 + 0.39)
L-aspartic acid	0.50	1.57	1.86 (1.86 + 0.00)
L-glutamic acid	2.27	3.52	4.36 (4.16 + 0.20)
Glycine	0.30	0.19	0.23 (0.23 + 0.00)
L-proline	1.76	2.07	2.45 (2.45 + 0.00)
L-serine	0.99	1.24	1.46 (1.46 + 0.00)
L-tyrosine	0.90	0.01	0.01 (0.01 + 0.00)

CAS: casein; fAA: free amino acids; GMP: glycomacropeptide. * GMP diet = BiPRO^®^ GMP + additional amino acids.
